# Utilization of orange peel waste for sustainable amino acid production by *Corynebacterium glutamicum*

**DOI:** 10.3389/fbioe.2024.1419444

**Published:** 2024-07-10

**Authors:** Nora Junker, Berna Sariyar Akbulut, Volker F. Wendisch

**Affiliations:** ^1^ Genetics of Prokaryotes, Faculty of Biology and Center for Biotechnology (CeBiTec), Bielefeld University, Bielefeld, Germany; ^2^ Department of Bioengineering, Marmara University, Istanbul, Türkiye

**Keywords:** *Corynebacterium glutamicum*, sustainable amino acid production, food waste upcycling, second generation feed stock, orange peel, agriculture sidestream

## Abstract

Oranges are the most processed fruit in the world–it is therefore apparent that the industrial production of orange juice generates large quantities of orange peel as a by-product. Unfortunately, the management of the orange peel waste leads to economic and environmental problems. Meanwhile, the use of sustainable raw materials for the production of bulk chemicals, such as amino acids, is becoming increasingly attractive. To address both issues, this study focused on the use of orange peel waste as a raw material for media preparation for the production of amino acids by engineered *Corynebacterium glutamicum*. *C. glutamicum* grew on pure orange peel hydrolysate (OPH) and growth was enhanced by the addition of a nitrogen source and a pH buffer. Inhibitory effects by the combination of high concentrations of OPH, (NH_4_)_2_SO_4_, and MOPS buffer in the wild-type strain (WT), were overcome in the tyrosine-producing engineered *C. glutamicum* strain AROM3. Genetic modifications that we identified to allow for improved growth rates under these conditions included the deletions of the vanillin dehydrogenase gene *vdh*, the ʟ-lactate dehydrogenase gene *ldhA* and the 19 genes comprising cluster cg2663-cg2686. A growth inhibiting compound present in high concentrations in the OPH is 5-(hydroxymethyl)furfural (HMF). We identified *vdh* as being primarily responsible for the oxidation of HMF to its acid 5-hydroxymethyl-2-furancarboxylic acid (HMFCA), as the formation of HMFCA was reduced by 97% upon deletion of *vdh* in *C. glutamicum* WT. In addition, we showed that growth limitations could be overcome by adjusting the media preparation, using a combination of cheap ammonia water and KOH for pH neutralization after acidic hydrolysis. Overall, we developed a sustainable medium based on orange peel waste for the cultivation of *C. glutamicum* and demonstrated the successful production of the exemplary amino acids ʟ-arginine, ʟ-lysine, ʟ-serine, ʟ-valine and ʟ-tyrosine.

## 1 Introduction

Citrus stands out as the most abundant fruit crop globally, with one-third of the harvest going to processing, e.g., to juice (Kumar et al., 2017). Typically, 50–60 wt% of processed citrus fruit remains as waste after juice extraction (Wilkins et al., 2007), contributing to more than 0.5 billion metric tons of agricultural waste from the fruit processing industry worldwide (Banerjee et al., 2017). In particular, orange (*Citrus sinensis*) stands out as the world’s most processed citrus fruit (Kumar et al., 2017), with a global output of more than 76 million metric tons annually. Brazil, China, and India are the main contributors to orange production, generating 17.1, 10.4, and 9.5 million metric tons per year, respectively, meanwhile Turkey also accounts for 2.4% of the global production (FAO, 2021).

The management of the fruit waste poses a significant challenge, giving rise to environmental concerns. Smaller facilities frequently resort to traditional disposal strategies such as landfilling (Girotto et al., 2015), which can cause soil and water pollution (Zema et al., 2018a), or incineration of these by-products (Torres-León et al., 2018). Both operations contribute to greenhouse gas emissions, while eliminating potential benefits of orange peels. Modern approaches include the use as animal feed or soil conditioner (Zema et al., 2018a). Dry orange peel is composed of approximately 14.4 wt% cellulose, 10.9 wt% hemicellulose, 1.3 wt% lignin, and 28.7 wt% pectin (Bicu and Mustata, 2011), making it an attractive source of fermentable sugars (Harmsen et al., 2010). In addition, it is also rich in nutrients required to support microbial growth, allowing it to be valorized as a feedstock for microbial production of valuable molecules (Bibi et al., 2016; Kuo et al., 2019).

Meanwhile, there is a high demand to reduce dependence on non-sustainable and fossil-based resources for the production of bulk chemicals. It is necessary to find sustainable and renewable raw materials that do not compete with food availability to achieve an environmentally responsible circular bioeconomy (Wendisch et al., 2022). In this context, the use of orange peels for biotechnological fermentations offers dual benefits: it provides a sustainable solution for managing the waste material and simultaneously offers economic benefits by converting waste into valuable bioproducts. This has been realized by extending the traditional ethanol fermentation of fruits to the fermentation of fruit wastes. Ethanol was produced from orange peel waste using a variety of organisms, including *Saccharomyces cerevisiae*, *Pichia kudriavzevii*, *Zymomonas mobilis*, *Mucor indicus* and *Escherichia coli*, achieving titers of 9–54 g/L (Grohmann et al., 1994; Wilkins, 2009; Oberoi et al., 2010; Lennartsson et al., 2012; Santi et al., 2015; Koutinas et al., 2016; Jha et al., 2019). Beyond ethanol, other biofuels were produced on orange peel waste, including methane, hydrogen, butanol, and fatty acid methyl esters (biodiesel) (Park et al., 2014; Joshi et al., 2015; Wikandari et al., 2015; Zema et al., 2018b; Abd-Alla et al., 2019). Additionally, the production of carboxylic acids using orange peel waste was demonstrated, such as citric acid [9.2 g/L by *Aspergillus niger* (Rivas et al., 2008)], D-lactic acid [48.9 g/L using *Lactobacillus delbrueckii* (De la Torre et al., 2019a)] or succinic acid [1.9 g/L using *Fibrobacter succinogenes* (Li et al., 2010)]. The product portfolio also encompasses the polymers xanthan (Mohsin et al., 2018) and polyhydroxybutyrate (PHB) (Davaritouchaee et al., 2023), in addition to volatile esters (Lalou et al., 2013) and few other products (Gervasi and Mandalari, 2024). Despite the rich nitrogen content of food wastes, which has been generally neglected, one of the biggest sectors of industrial biotechnology (Wendisch et al., 2022), the production of the nitrogenous amino acids, has not yet received attention regarding the use of orange peel wastes.

Amino acid production is a multi-million-ton business that still uses food- and feed-competitive raw materials. In general, amino acids are used for a wide range of purposes, especially in the food and feed, but also in the pharmaceutical industries. ʟ-Lysine is one of the leading biotechnological products with an annual production of about 2,600,000 metric tons (Wendisch, 2020). It has a well-established role as a feed additive and has potential as a precursor for biopolymers (Eggeling and Bott, 2015). Since ʟ-valine, as well as ʟ-lysine, is among the essential amino acids for higher organisms, it is used in dietary products and cosmetics, but also as a precursor for antibiotics or herbicides (Oldiges et al., 2014). ʟ-Arginine has particular applications in the pharmaceutical industry, e.g., it can be used to lower blood pressure (Siani et al., 2000). This beneficial effect is attributed to arginine being a precursor of nitric oxide (NO), a key component of endothelium-derived relaxing factor (Appleton, 2002). The amino acids ʟ-serine and ʟ-tyrosine are important precursors in the pharmaceutical industry as their derivatives have a wide range of applications. One common application is the treatment of Alzheimer’s disease, for which both phosphatidylserine (Zhang et al., 2015) and the tyrosine derivative ʟ-DOPA (Dorszewska et al., 2014) can be used.

The leading position for amino acid production is occupied by *Corynebacterium glutamicum*. With a well-developed genetic toolbox and decades of metabolic engineering exploration (Lee and Wendisch, 2017), *C. glutamicum* has also been engineered for the production of a plethora of high-value active ingredients for food, feed, human health, and well-being (Wolf et al., 2021). Further engineering of *C. glutamicum* has been accomplished for feedstock flexibility (Wendisch et al., 2016), making it suitable for the cultivation on agricultural waste hydrolysates. This is further supported by its ability to detoxify pretreatment derived inhibitors, such as furfural, HMF, and 4-hydroxybenzaldehyde (Wang et al., 2018).

Harnessing the potential of orange peel waste in biotechnological fermentations, particularly for amino acid production using *C. glutamicum*, represents a promising avenue. This approach not only addresses the environmental challenges associated with citrus waste, but also meets the increasing market demand for sustainable biotechnological production processes, steering away from food-competitive raw materials. This study dealt with the exploitation of orange peels as an alternative renewable raw material for the production of amino acids by employing metabolically engineered *C. glutamicum* strains.

## 2 Materials and methods

### 2.1 Source of orange peel and pretreatment

Orange peel was kindly provided by GE-TA Tarım ve Gıda San. Tic. A.Ş. The peel samples were dried in an oven at 50°C for 2–3 days and then grounded in a 1 L Waring commercial blender. The prepared orange peel powder was stored at −20°C to avoid contamination.

### 2.2 Acidic hydrolysis

For the preparation of orange peel hydrolysates (OPH), 25 g of dried orange peel powder was mixed with 200 mL of 2 vol% H_2_SO_4_. The mixture was autoclaved at 121°C for 50 min (Pakalın et al., 2023). After centrifugation at 4,000 rpm for 30 min, the pH of the supernatant was adjusted to 7 using 10 M KOH, unless otherwise stated. For the later developed OPH-NH_3_ solution, ammonia water (25 vol%) was added to the hydrolysate to a final concentration of 375 mM NH_3_ prior to pH neutralization with 10 M KOH. An 80 vol% OPH-NH_3_ solution thus contains OPH with additional 300 mM of NH_3_. The hydrolysate was centrifuged again (4,000 rpm, 30 min) prior to sterile filtration. After storage for at least 2 weeks at room temperature or 30°C, the hydrolysate was used for cultivation experiments.

### 2.3 Quantification of amino acids, carbohydrates, and furfural derivatives

Prepared hydrolysates were analyzed for their carbohydrates, HMF derivatives and amino acids using a high-performance liquid chromatography (HPLC) system (1200 series, Agilent Technologies Deutschland GmbH, Böblingen, Germany). Prior to measurement, OPH samples were centrifuged (14,000 rpm, 20 min) and diluted with water.

Carbohydrates, HMF, and HMFCA were separated with an organic acid resin pre- and main column (Aminex, 40 × 8 mM and 300 × 8 mM, respectively, 10 μm particle size, 25 Å pore diameter, CS Chromatographie Service GmbH) under isocratic conditions with 5 mM H_2_SO_4_ as the mobile phase at a flow rate of 0.8 mL/min and 60°C column temperature for 35 min. A refractive index detector (RID G1362A, 1200 series, Agilent Technologies) was used to detect carbohydrates, while a diode array detector (DAD G1315B, 1200 series, Agilent Technologies) was used to quantify HMF at 284 nm and HMFCA at 260 nm.

Amino acids with a primary amine were measured via derivatization with *ortho*-phthaldialdehyde (OPA). Prior to measurement, the centrifuged and diluted samples were supplemented with cadaverine to a final concentration of 0.1 mM as an internal standard. The same procedure was carried out with amino acid standard solutions, which were prepared in concentrations of 0.05 mM–1.5 mM. The samples were automatically mixed with the derivatization reagent OPA by the autosampler module prior to injection. Amino acids were separated using a reversed phase pre- and main column (LiChrospher 100 RP18 EC-5 μ, 40 mM × 4.6 mM and 125 mM × 4.6 mM, respectively, CS-Chromatographie Service GmbH, Langerwehe, Germany). Due to the derivatization, a fluorescence detector with an excitation wavelength of 230 nm and an emission wavelength of 450 nm could be used for detection of the fluorescent derivatives. A gradient with (A) 0.25 vol% sodium acetate (pH 6.0) and (B) methanol as the mobile phase was applied, starting with 20 vol% B and a flow rate of 0.7 mL/min, 1 min 38 vol% B, 3 min 46 vol% B, 5 min 50 vol% B with a decrease of the flow rate to 0.5 mL/min, 5.5 min 51 vol% B, 8.3 min 53 vol% B, 9 min 54 vol% B, 12 min 58 vol% B with the flow rate increased to 0.8 mL/min, 16 min 65 vol% B with the flow rate increased to 1 mL/min, 19 min 80 vol% B with the flow rate increased to 1.2 mL/min, 19.5 min 90 vol% B, 20.5 min reduction of B to 20 vol% B, and maintaining this condition for 2 min.

The amino acid proline was derivatized with 9-fluorenylmethyl chloroformate (FMOC) as secondary amines cannot be derivatized with OPA. Therefore, 20 μL of the sample was mixed with 60 μL borate buffer (0.5 M, pH 9.0) containing cadaverine as an internal standard by vortexing. After adding 80 µL of FMOC reagent (30 mM, dissolved in acetone), the samples were vortexed and incubated for 45 s. Remaining FMOC was quenched by adding 100 µL of an isoleucine solution (80 mM, dissolved in 0.5 M borate buffer, pH 7), followed by vortexing and incubation for 45 s. Finally, 538 µL of dilution buffer (30 vol% 0.05 M sodium acetate, pH 4.2, and 70 vol% acetonitrile) was added. The measurement was done using the same reversed-phase pre- and main column, and fluorescence detector as described for the measurement with OPA derivatization, however with the excitation and emission wavelengths of 263 nm and 310 nm, respectively. The mobile phases were (A) 50 mM sodium acetate (pH 4.2) and (B) acetonitrile with an applied gradient starting with 38 vol% B (0 min) and increase as follows: 5 min 38 vol% B, 12 min 57 vol% B, 14 min 76 vol% B, 15 min 76 vol% B, and 18 min 38 vol% B.

All analytes (carbohydrates, HMF derivatives, and amino acids) were quantified using several appropriate dilutions of the respective compound as a standard.

### 2.4 Identification of HMF degradation product by mass spectrometry

The HMF degradation product 2,5-bis(hydroxymethyl)furan (BHMF) was identified using gas chromatography-mass spectrometry (GC-MS). Therefore, 300 µL of the sample was mixed with the same volume of ethyl acetate for extraction by vortexing. The organic phase was retained after centrifugation (14,000 rpm, 10 min) and analyzed by GC-MS measurement using a TraceGC gas chromatograph (Thermo Scientific, Waltham, MA, United States) and ISQ ion trap mass spectrometer (Thermo Scientific, Waltham, MA, United States). 5 μL of sample was injected at a split ratio of 20:1. Separation was achieved using a TraceGOLD TG-5MS (30 m × 0.25 mM, film thickness 0.25 μm, Thermo Scientific, Waltham, MA, United States) and helium as carrier gas at a constant flow rate of 0.6 mL/min. The temperature gradient in the oven was set to 120°C for 5 min, heated to 180°C at a rate of 3 °C/min and further increased to 220 °C at 1.2 °C/min. Meanwhile, temperatures for injector, interface and ion source were set to 250°C, 250°C, and 220°C, respectively. After 5 min, mass spectra were recorded with a scan range of m/z 30–550 and evaluated using the Xcalibur software version 2.0.7 (Thermo Scientific, Germany).

### 2.5 Determination of the composition of OPH-NH_3_ solution

The prepared OPH-NH_3_ solution was sent to Eurofins Agraranalytik Deutschland GmbH (Jena, Germany) for determination of organic matter (gravimetry; DIN EN 12879 (S3a): 2001-02; Loss on ignition at 550°C), nitrogen (combustion, DIN EN 16168: 2012-11), phosphorus, sulfur, potassium, calcium, zinc, boron, copper, manganese, and magnesia (ICP-OES; DIN EN 16174: 2012-11; DIN EN ISO 11885:2009-09), and molybdenum (ICP-MS(n); DIN EN 16174: 2012-11; DIN EN ISO 17294-2 (E29): 2017-01). The carbon content was calculated using the assumption that organic matter contains about 50 wt% carbon (Pribyl, 2010).

### 2.6 Microorganisms and cultivation conditions

All strains used in this study are derived from *C. glutamicum* ATCC 13032 and are listed in Table 1. The plasmid pECXT99A-*crimson* was used to transform AROM3 by electroporation, as described previously (van der Rest et al., 1999; Eggeling and Bott, 2005). For growth and production experiments, *C. glutamicum* strains were cultivated overnight in 20 mL Lysogeny Broth medium (LB; 10 g/L tryptone, 10 g/L sodium chloride, and 5 g/L yeast extract) at 120 rpm and 30°C. The overnight culture was used to inoculate a second LB preculture to a cell dry weight concentration (CDW) of 0.18 g/L. LB medium containing 20 vol% of OPH was used for the second preculture when the cultivated cells were used for experiments in OPH. After cultivation for 5–6 h, the preculture was centrifuged (4,000 rpm, 7 min) and used for inoculation of the main culture to an initial CDW of 0.35 g/L. CGXII minimal medium (Eggeling and Bott, 2005) supplemented with glucose was used as control. Antibiotics (25 μg/mL kanamycin, and 5 μg/mL tetracycline) as well as 1 mM isopropyl-β-D-1-thiogalactopyranoside (IPTG) to induce gene expression, were added to pre- and main cultures if required. Further supplements, depending on the requirements of the cultivated strain, are listed in Table 1. For cultivation of *C. glutamicum* VAL1 in OPH, the isoleucine present in OPH was included in the calculation of its final concentration, whereas for cultivation of *C. glutamicum* AROM3, phenylalanine was supplemented only for growth in CGXII minimal medium, but not for growth in OPH.

**TABLE 1:** Plasmids and bacterial strains used in this study and their required supplements.

Plasmid/strain	Relevant characteristics	Antibiotic resistance	Supplements	References
pECXT99A-*crimson*	*C. glutamicum/E. coli* expression shuttle vector (Tet^R^, P_*trc*_, *lacI* ^*q*^, pGA1 *oriV*_*C.g*._) expressing *crimson*	Tetracycline	IPTG	Sgobba et al. (2018)
*C. glutamicum* WT	*C. glutamicum* ATCC 13032	—	—	ATCC
*C. glutamicum* WT (pECXT99A-*gfp*_UV_)	—	Tetracycline	IPTG	Henke et al. (2021)
∆*vdh*	(Δcg2953)	—	—	Lessmeier et al. (2013)
∆*ldhA*	(Δcg3219)	—	—	Unthan et al. (2015)
N646	Δcg2663–cg2673	—	—	Unthan et al. (2015)
N616	N646 Δcg2674–cg2686	—	—	Unthan et al. (2015)
C1*	N616 ΔCGP123 ΔISCg1 ΔISCg2 Δcg2312–cg2322 Δcg2621–cg2643 Δcg2755–cg2760 Δcg3102–cg3111 Δcg0635–cg0646 Δcg0704–cg0748 Δcg0822–cg0845 Δcg1018–cg1033 Δcg1172–cg1213 Δcg1291–cg1305	—	—	Baumgart et al. (2018)
ARO02	C1* ∆*ldhA* ∆*vdh*::P_*ilvC*_-*aroG*_*Ec*_^D146N^	—	—	Walter et al. (2020)
AROM1	ARO02 *trpE* ^TTG^	—	—	Kurpejović et al. (2023)
AROM3	AROM1 *pheA* ^TTG^	—	0.5 mM Phenylalanine	Kurpejović et al. (2023)
AROM3 (pECXT99A-*crimson*)	—	Tetracycline	0.5 mM Phenylalanine, IPTG	This work
ARG5	MB001 Δ*argFRG* (pVWEx1-*argGFB* ^A49V,M54V^)	Kanamycin	IPTG	Jensen et al. (2015)
VAL1	Δ*ilvA* Δ*panBC* (pJC1-*ilvBNCD*)	Kanamycin	3 μM Pantothenate, 3.4 mM Isoleucine	Lange et al. (2003)
GRLys1 Δ*sugR* Δ*ldhA*	Δ*pck*, *pyc* ^P458S^, *hom* ^V59A^, 2 copies of *lysC* ^T311I^, 2 copies of *asd*, 2 copies of *dapA*, 2 copies of *dapB*, 2 copies of *ddh*, 2 copies of *lysA*, 2 copies of *lysE*, in-frame deletion of prophages CGP1 (cg1507–cg1524), CGP2 (cg1746–cg1752) and CGP3 (cg1890–cg2071), Δ*sugR* Δ*ldhA*	—	—	Pérez-García et al. (2016)
SER1	Δ*pabABC* Δ*sdaA* (pEC-T18mob2-*serA* ^fbr^ *CB*)	Tetracycline	0.1 mM Folate	Stolz et al. (2007)

Growth and production experiments were carried out in 48-well FlowerPlates (m2p-labs, Baesweiler, Germany) with a filling volume of 1 mL per well at 1,100 rpm and 30°. Growth was followed by automated backscatter measurements by the BioLector microcultivation system (m2p-labs, Baesweiler, Germany). In addition, the optical densities at 600 nm (OD_600_) were determined with a V-1200 Spectrophotometer (VWR, Radnor, PA, United States) and biomass concentrations were determined according to the correlation CDW = 0.353 × OD_600_ (g/L) (Kiefer et al., 2004). Each cultivation was performed in triplicate.

### 2.7 Fluorescence analysis by flow cytometry and fluorescence microscopy

To distinguish two *C. glutamicum* strains, WT and AROM3, in a co-cultivation approach, the expression of fluorescent marker proteins was used. Quantification of the ratio of the two strains was done by flow cytometry (flow cytometer Gallios™, Beckman Coulter, Krefeld, Germany). Besides the samples from the co-cultivation experiment, the same strains in monoculture and a strain without fluorescent protein expression were used as controls. Cell samples were washed twice with TN-buffer after cultivation and diluted to a CDW of approximately 0.18 g/L. Per sample, 20,000 events were analyzed using a blue solid-state laser at an excitation wavelength of 405 nm to monitor fluorescence via the forward scatter (FSC) and side scatter (SSC) signals. Band-pass filters for 525/40 nm and 660/20 nm were used to detect the GFP and Crimson signals, respectively. In addition, the cell samples were visualized by fluorescence microscopy. The washed cell samples were mixed with agarose solution (10 g/L dissolved in TN buffer) for fixation on the microscope slides and observed at ×60 magnification in immersion oil with a Keyence BZ-X810 fluorescence microscope (Keyence Deutschland GmbH, Neu-Isenburg, Germany). GFP/FITC/A488 and RFP/A555/P filters were used to create overlay images with GFP and Crimson visualization using the Keyence software.

## 3 Results

### 3.1 Characterization of orange peel hydrolysate as a medium for cultivation of *C. glutamicum*

**FIGURE 1:**
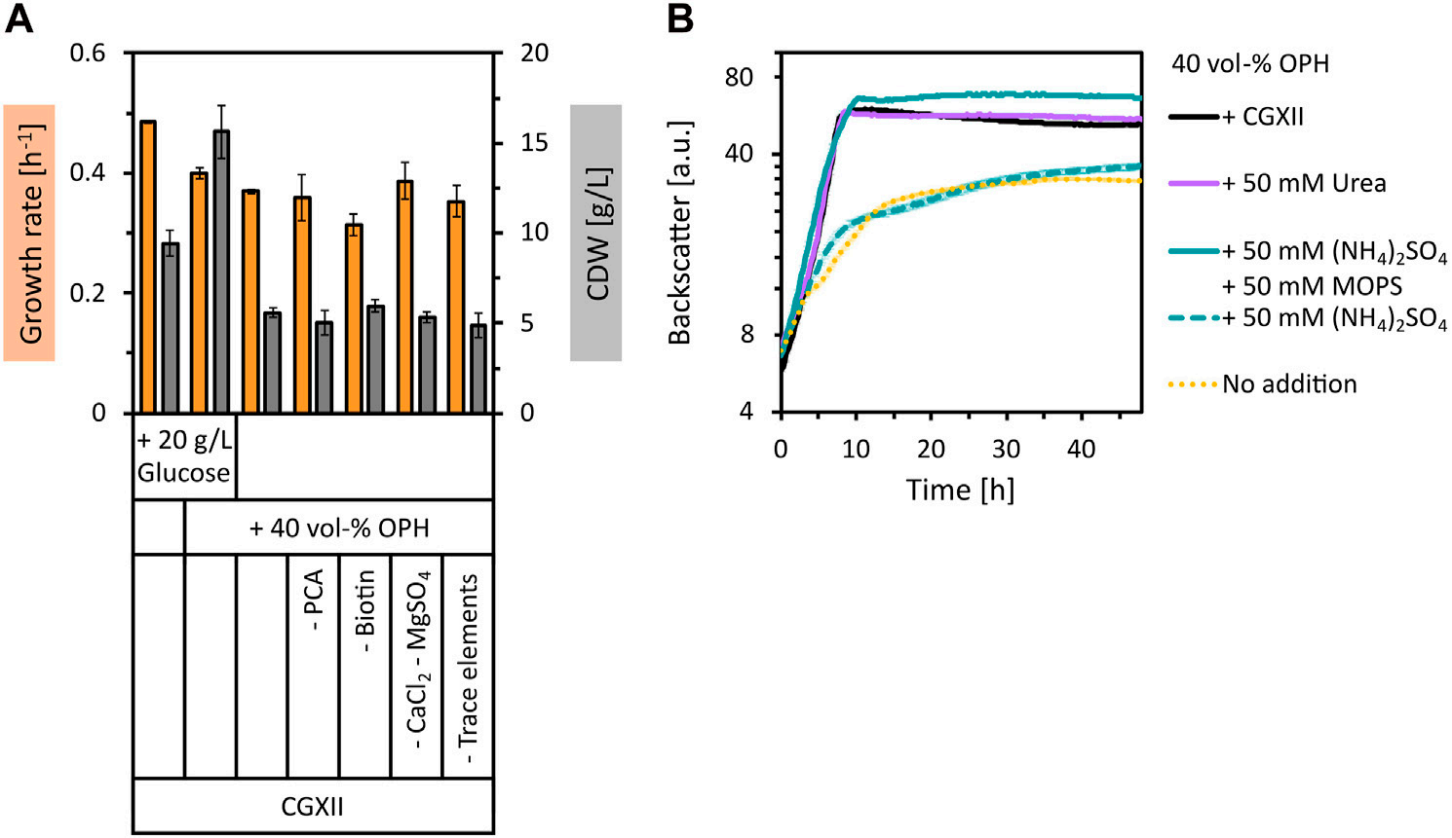
Growth of *C. glutamicum* with supplementation of OPH. **(A)** Comparison of maximum specific growth rates (orange bars) and final biomass concentration (CDW, grey bars) for cultivation in CGXII medium with and without 20 g/L glucose and 40 vol% OPH and in CGXII +40 vol% OPH leaving out different CGXII components [PCA, biotin, CaCl_2_ and MgSO_4_, trace elements (FeSO_4_, MnSO_4_, ZnSO_4_, CuSO_4_, NiCl_2_)]. **(B)** Comparison of growth in 40 vol% OPH with addition of CGXII (black line) or with addition of nitrogen sources [urea (purple line), (NH_4_)_2_SO_4_ (dashed, turquoise line) or (NH_4_)_2_SO_4_ and MOPS (turquoise line)]. *C. glutamicum* WT (pECXT99A-*gfp*_UV_) was cultivated in the BioLector microcultivation system. Values and error bars represent means and standard deviations of triplicate cultivations.

To use orange peel waste as a feedstock for biotechnological fermentation processes, the polymer structures must be broken down by hydrolysis. This was achieved by acid hydrolysis of dried orange peel at 121°C for 50 min using 2 vol% of H_2_SO_4_, followed by pH neutralization with KOH. The sugars with the highest concentration in the prepared OPH were glucose (17.8 ± 2.1 g/L), xylose (11.7 ± 1.2 g/L), arabinose (7.5 ± 0.5 g/L), and galacturonic acid (4.2 ± 0.5 g/L). The total amount of amino acids quantified was 1.61 ± 0.06 g/L, with aspartate (0.71 ± 0.25 g/L), proline (0.32 ± 0.05 g/L), serine (0.11 ± 0.05 g/L) and alanine (0.09 ± 0.05 g/L) having the highest concentrations (see Supplementary Table S1 for a complete list of amino acid concentrations in OPH). Besides sugars and amino acids, compounds with inhibitory properties towards microorganisms such as weak organic acids, furan derivatives, and phenolic compounds are also generally formed or released during hydrolysis of lignocellulosic materials (Jönsson et al., 2013). This is also true for the obtained OPH, as, e.g., the furfural derivative HMF was quantified with 2.29 ± 0.03 g/L. The tolerance of *C. glutamicum* WT towards 40 vol% OPH was tested by supplementing it to CGXII minimal medium containing 20 g/L glucose. The addition of OPH resulted in an increase in biomass formation of *C. glutamicum* by more than 1.8-fold compared to the control (Figure 1A). This increase in biomass indicates that *C. glutamicum* not only tolerated the OPH components, but also utilized nutrients in the OPH for biomass formation. Subsequently, we tested the use of OPH as the sole carbon and energy source in minimal medium. In CGXII medium with 40 vol% OPH (therefore containing 7.1 ± 0.8 g/L glucose), *C. glutamicum* grew to a biomass concentration of 5.6 ± 0.3 g/L CDW (Figure 1A), demonstrating the use of OPH as the sole carbon source.

Next, it was tested whether OPH could eliminate the need for CGXII media components. Indeed, neither PCA, biotin, CaCl_2_, MgSO_4_, nor trace elements were required, when 40 vol% OPH was supplemented (Figure 1A). Subsequently, growth of *C. glutamicum* in pure OPH (diluted to 40 vol%) without any CGXII media components was tested. Surprisingly, growth to a biomass concentration of 3.6 ± 0.2 g/L CDW was observed (Figure 1B). However, compared to growth in CGXII medium plus 40 vol% OPH, the biomass formation and the growth rate were reduced, indicating a limitation. To test whether the nitrogen source was the limiting factor, supplementation of the 40 vol% OPH with urea was tested as a medium. Indeed, the addition of 50 mM urea to the OPH restored the growth behavior of *C. glutamicum* to that observed in CGXII medium with 40 vol% OPH (Figure 1B). Supplementing 50 mM (NH_4_)_2_SO_4_ did not have the same positive effect as the addition of urea. However, when (NH_4_)_2_SO_4_ was combined with a buffer system (50 mM MOPS) to maintain the pH value, the growth limitation was overcome. To formulate a cost-effective and sustainable medium for cultivation processes with *C. glutamicum*, (NH_4_)_2_SO_4_ was chosen over urea due to its lower cost as a nitrogen source. Taken together, 40 vol% OPH supplemented with 50 mM (NH_4_)_2_SO_4_ and buffered with 50 mM MOPS was shown to be a suitable growth medium for *C. glutamicum* WT.

### 3.2 OPH-based media for production of nitrogenous compounds by *C. glutamicum*

**FIGURE 2:**
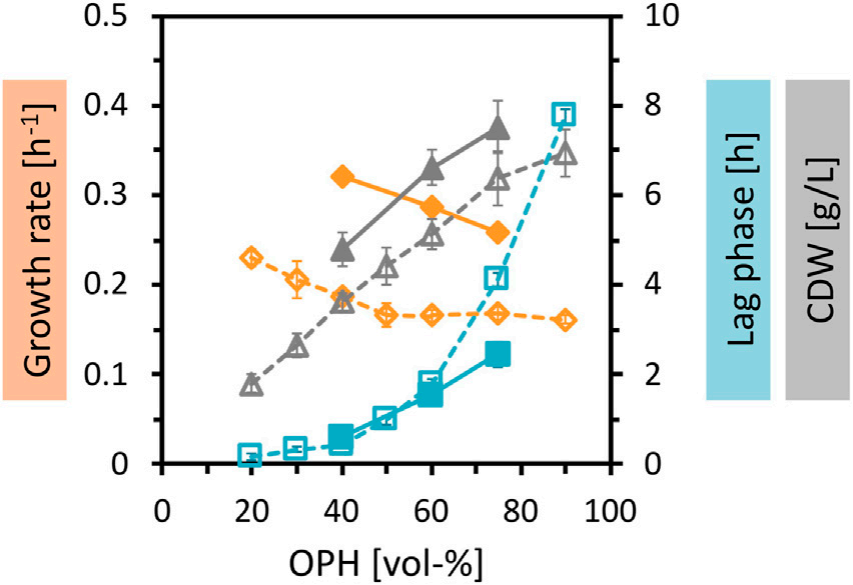
Growth of *C. glutamicum* in different concentrations of OPH. *C. glutamicum* WT (pECXT99A-*gfp*_UV_) was cultivated in different dilutions of OPH (20–90 vol%) (empty symbols and dotted lines) and in 40–75 vol% OPH supplemented with 50 mM (NH_4_)_2_SO_4_ and 50 mM MOPS (filled symbols and solid lines). The cultivation was performed in the BioLector microcultivation system. Maximum specific growth rates (orange), lag phases (blue), and CDW (gray) represent the means of triplicate cultivations with their standard deviations depicted as error bars.

Fermentative production of amino acids requires sufficient supply of carbon and nitrogen. To achieve higher biomass concentrations and higher production, the nutrient content of the OPH medium was increased. First, the OPH concentration was gradually increased up to 90 vol%, which successfully improved biomass formation (Figure 2). Second, 50 mM (NH_4_)_2_SO_4_ was added as the nitrogen source with 50 mM MOPS as a buffer. As shown before, the supplementation of (NH_4_)_2_SO_4_ and MOPS improved the growth parameters, leading to an increase in growth rate, a decrease of lag phase, and an increase in total biomass formation.

**FIGURE 3:**
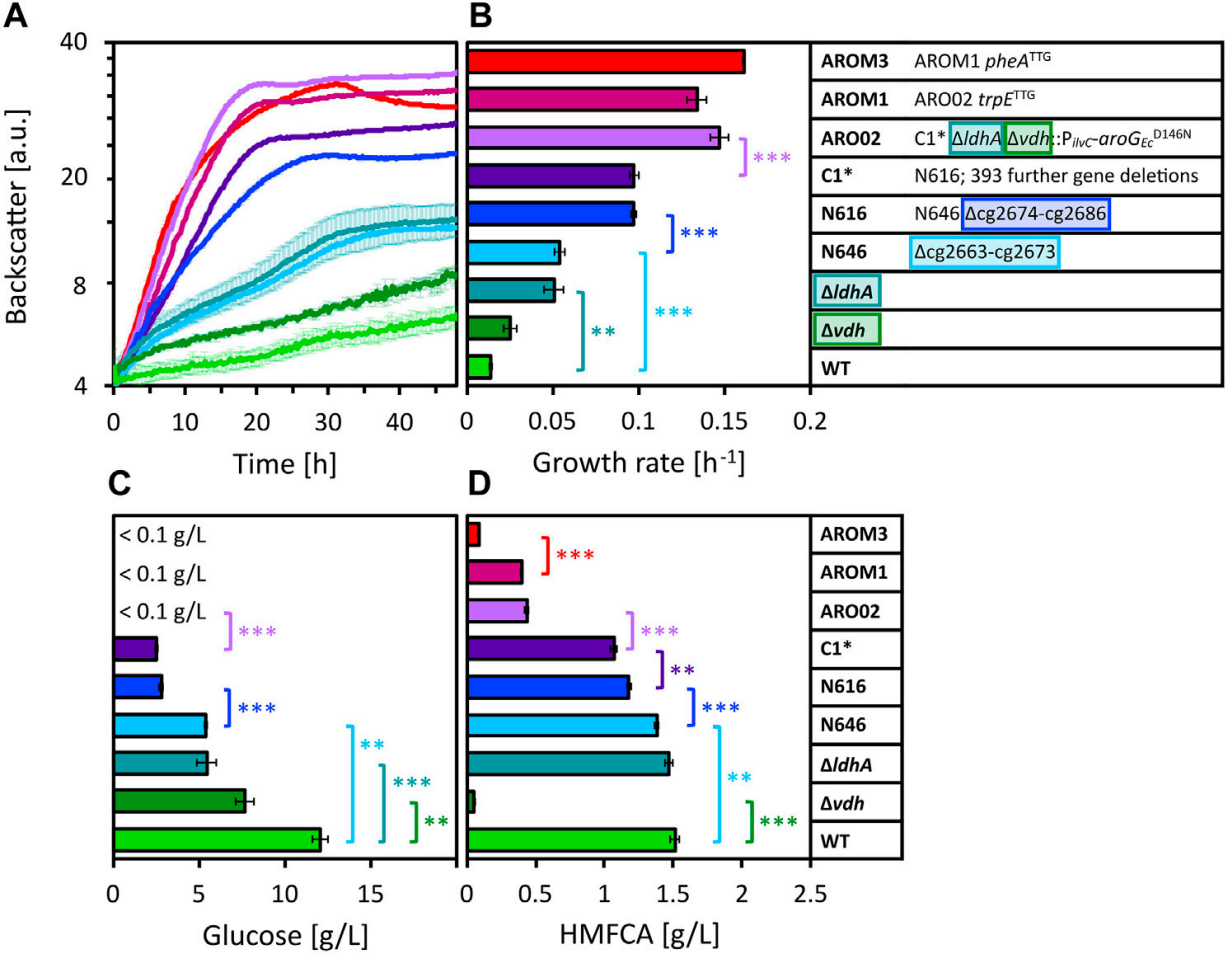
Growth of different WT-derived *C. glutamicum* strains in 80 vol% OPH with 150 mM (NH_4_)_2_SO_4_ and 200 mM MOPS. Growth was monitored by measuring the backscatter in the BioLector microcultivation system **(A)** and maximum specific growth rates were calculated based on the backscatter values **(B)**. Residual glucose **(C)** was measured after cultivation, as well as HMFCA concentration **(D)**. Values and error bars represent means and standard deviations of triplicate cultivations. Statistical significance was determined by two-sided unpaired Student’s t-tests and depicted for *p*-value of *p* < 0.01 (**) and <0.001 (***).

When the nitrogen supply was further increased by adding 150 mM (NH_4_)_2_SO_4_ to 80 vol% OPH, growth was strongly inhibited. This occurred with both buffering approaches, i.e., 50 mM MOPS (here, the pH shifted to 5 during cultivation, data not shown) or 200 mM MOPS (Figure 3, green line). To tackle this issue, two different approaches were addressed: the first focused on the growth ability of the strain by testing strains with different genetic modifications, and the second targeted the OPH medium by adjusting its preparation.

#### 3.2.1 Identification of gene deletions relevant for overcoming growth inhibition

Besides *C. glutamicum* WT, the genome-reduced chassis strain C1* is often used in metabolic engineering for the production of valuable compounds. Strain C1* is derived from *C. glutamicum* by 412 deletions (ΔCGP123 ΔISCg1 ΔISCg2 Δcg2312–cg2322 Δcg2621–cg2643 Δcg2755–cg2760 Δcg3102–cg3111 Δcg0635–cg0646 Δcg0704–cg0748 Δcg0822–cg0845 Δcg1018–cg1033 Δcg1172–cg1213 Δcg1291–cg1305 Δcg2663–cg2686), but it grows on glucose minimal medium as well as the parental *C. glutamicum* WT (Baumgart et al., 2018). When cultivated in OPH with high C and N source loadings (80 vol% OPH, 150 mM (NH_4_)_2_SO_4_, 200 mM MOPS), C1* grew faster and to higher biomass concentrations than WT. Thus, the deletion of one or more of the 412 genes present in WT, but absent from C1* was beneficial for growth in OPH at high C and N loadings. We recently engineered the C1*-based *C. glutamicum* strain AROM3 (genetic background: C1* ∆*ldhA* ∆*vdh*::P_*ilvC*_-*aroG*_*Ec*_^D146N^ *trpE* ^TTG^ *pheA* ^TTG^) for efficient tyrosine production. Surprisingly, AROM3 grew even better than C1* in OPH at high C and N loadings. This prompted us to compare several strains related to WT, C1* and AROM3 (Figure 3).

The strain with the single gene deletion of *ldhA*, encoding the ʟ-lactate dehydrogenase (Inui et al., 2004), exhibited a significantly increased growth rate compared to WT (Figure 3B). The deletion Δcg2663-cg2673 (strain N646) also caused a significant enhancement in the growth rate relative to the parental WT strain. Compared to strain N646, eleven additional genes (cg2674–cg2686) are deleted in *C. glutamicum* strain N616 resulting in an almost doubled growth rate. Further 393 genes are deleted in C1*, yet these deletions have not led to an increased growth rate. The residual glucose concentrations after cultivation underline the growth limitations of the different strains (Figure 3C). Considering these concentrations reveals that also the single gene deletion of *vdh*, encoding the vanillin dehydrogenase (Ding et al., 2015), relieved part of the growth inhibition, allowing for higher glucose consumption. The aforementioned observations were confirmed by residual glucose concentrations, namely, that also the deletions of *ldhA*, cg2663-cg2673 and cg2674–cg2686 relieved part of the growth inhibition. Taken together, four deletions (Δ*vdh*, Δ*ldhA*, Δcg2663–cg2673, and Δcg2674–cg2686) improved growth of the parental strain *C. glutamicum* WT (a list of genes and the functions of their encoded proteins can be found in the Supplementary Table S2). When these deletions were combined (as shown in ARO02), glucose was completely consumed. This additive positive effect was also apparent in the growth rate, which was significantly increased for ARO02 compared to its parental strain C1*. ARO02 furthermore carries *aroG*_*Ec*_^D146N^ encoding the feedback resistant 3-deoxy-D-arabino-heptulosonate-7-phosphate (DAHP) synthase from *E. coli* (Kikuchi et al., 1997), which is homologous to *C. glutamicum*’s native DAHP synthases encoded by *aroF* and *aroG*. The genetic modifications that distinguish ARO02 from the final tyrosine producer strain AROM3, namely, the translation start codon exchanges in the genes *pheA* and *trpE* from ATG to the less preferred TTG, did not affect the growth rate in the same manner as the previously mentioned modifications.

One growth inhibitor detected in OPH is HMF. It has been described that *C. glutamicum*, unlike *E. coli*, not only reduces furfural and HMF to their respective alcohols, but also oxidizes them to their respective acid forms for detoxification (Tsuge et al., 2014; Zhou et al., 2019). Indeed, we found that HMF (with an initial concentration of 1.8 g/L) was barely present after cultivation (<0.13 g/L for all samples) and instead detected the alcohol and the acid form of HMF, namely, BHMF and HMFCA, respectively (exemplary chromatograms are depicted in Supplementary Figures S1, S2). The formation of these two compounds BHMF and HMFCA was further confirmed by cultivation of *C. glutamicum* WT and AROM3 in CGXII minimal medium with supplementation of 10 mM HMF (data not shown). Quantification of HMFCA after cultivation in OPH revealed that, notably, the concentration of HMFCA was drastically reduced in strains lacking *vdh* (Figure 3D).

To sum it up, the deletions of *vdh*, of *ldhA*, of at least one gene of Δcg2663–cg2673, and of at least one gene of cg2674–cg2686 improved growth in OPH at high C and N loadings, and their effects were additive.

**FIGURE 4:**
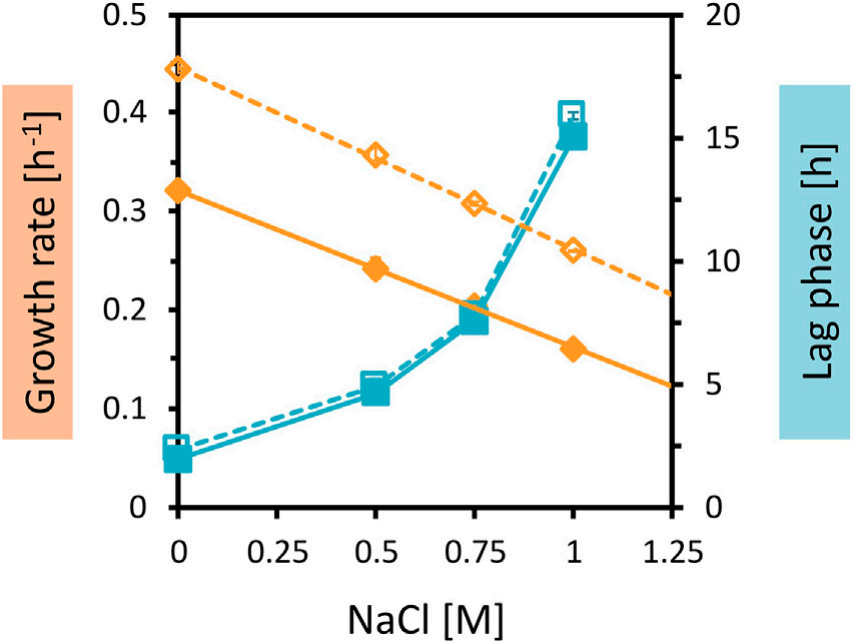
Growth parameters of *C. glutamicum* WT and AROM3 cultivated with addition of increasing NaCl concentrations. The two strains WT (dotted line and empty symbols) and AROM3 (solid line and filled symbols) were grown in CGXII medium plus 20 g/L glucose with supplementation of 0–1 M NaCl in the BioLector microcultivation system. Maximum specific growth rates (orange diamonds) and corresponding regression lines (orange), as well as the lag phases (blue squares) are plotted as a function of NaCl concentration. Values and error bars represent means and standard deviations of triplicate cultivations.

While the genetic modifications were identified, their physiological effects on growth in OPH with high C and N loadings could not be deduced from the annotations of the genes. Since *C. glutamicum* WT was able to grow in a medium with high OPH loading, and also in medium containing 40 vol% OPH and 150 mM (NH_4_)_2_SO_4_ with 200 mM MOPS, but not in the combination of both (80 vol% OPH and 150 mM (NH_4_)_2_SO_4_ with 200 mM MOPS), osmotic stress was considered to be a possible factor. To test the response of *C. glutamicum* WT and AROM3 to osmotic and salt stress, the two strains were cultivated in CGXII medium with increasing NaCl concentrations (Figure 4).

The very comparable increases in the lag phases with rising NaCl concentrations and small differences in the concentrations leading to half-maximal growth rates (K_i_ values of about 1 M for AROM3 and about 1.2 M for WT) (Figure 4) make it unlikely that a difference in osmotic tolerance between AROM3 and WT was responsible for the observed strain-specific growth pattern with OPH (Figure 3). As expected from the tolerance to NaCl, addition of sorbitol, which imposes osmotic but not salt stress, or addition of Na_2_SO_4_ to test for possible inhibition by sulfate, affected both *C. glutamicum* WT and AROM3 in a comparable manner (Supplementary Figure S2).

**FIGURE 5:**
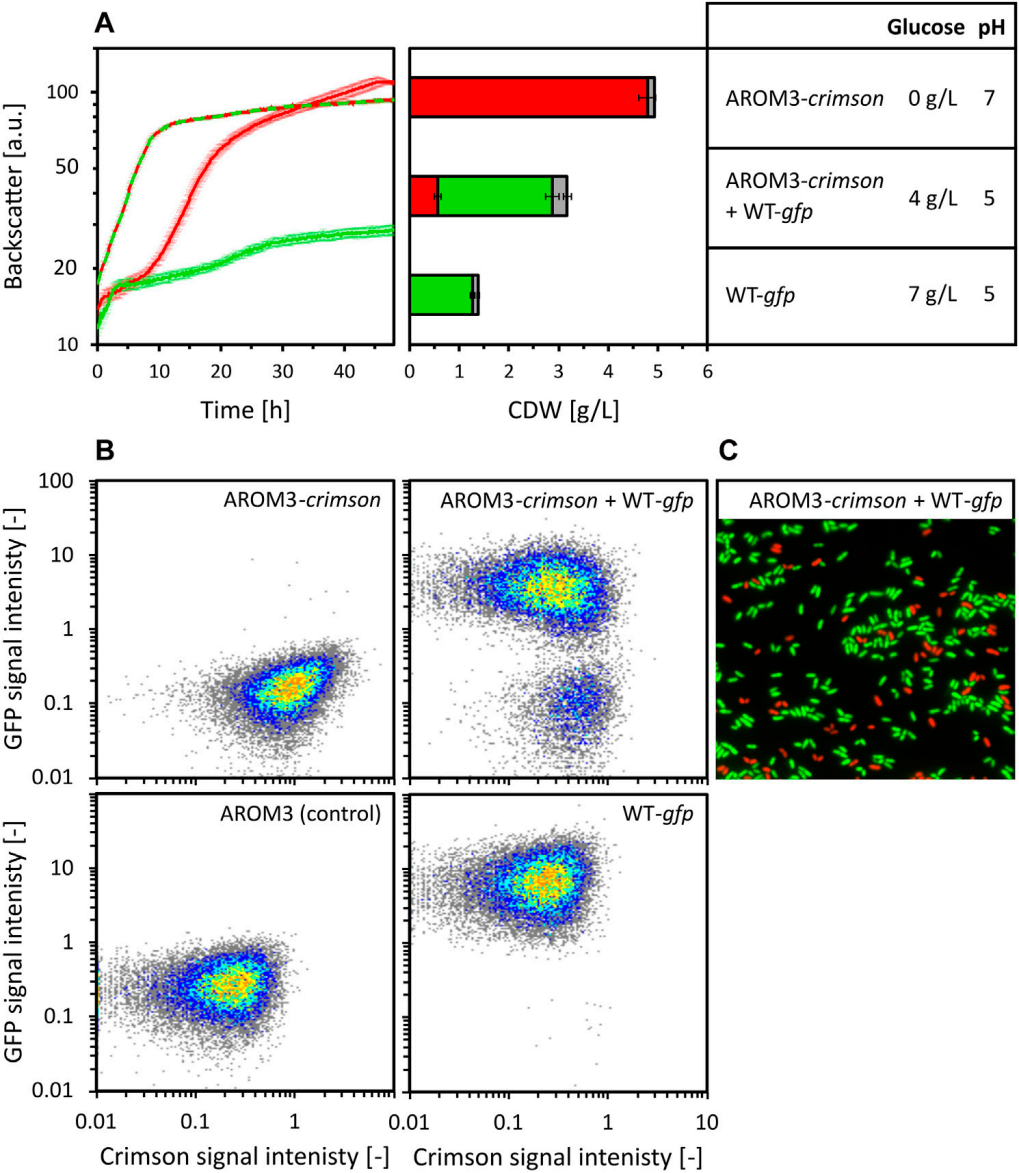
Co-cultivation of *C. glutamicum* WT (pECXT99A-*gfp*_UV_) (shown as WT-*gfp*) and AROM3 (pECXT99A-*crimson*) (shown as AROM3-*crimson*). The strains were cultivated separately (WT-*gfp* depicted in green and AROM3-*crimson* depicted in red) with a starting CDW of 0.35 g/L and in co-cultivation (depicted in red/green) with a total starting CDW of 0.7 g/L in 80 vol% OPH supplemented with 150 mM (NH_4_)_2_SO_4_, 200 mM MOPS. Backscatter was measured by the BioLector microcultivation system and residual glucose concentrations and pH values were measured for the supernatants after 48 h of cultivation **(A)**. The ratio of Crimson and GFP containing cells was determined by fluorescence analysis via flow cytometry [depicted for CDW in red and green, respectively, while gray shows the ungated events, (**A**, middle)]. Values and error bars represent means and standard deviations of triplicate cultivations, the standard deviation of glucose concentration was <1 g/L. The corresponding scatter plots **(B)** are shown for one of each triplicate measurements: AROM3 without fluorescent marker gene as negative control (lower left corner), *C. glutamicum* WT (pECXT99A-*gfp*_UV_) (upper left corner), AROM3 (pECXT99A-*crimson*) (lower right corner), and consortia of *C. glutamicum* WT (pECXT99A-*gfp*_UV_) + AROM3 (pECXT99A-*crimson*) (upper right corner). The ratios were confirmed by fluorescence microscopy overlays, as exemplarily shown for one of the consortia samples **(C)**.

To test whether the formation or utilization of a growth inhibitory compound by one strain, but not the other, causes the different growth behavior of WT and AROM3, a co-cultivation of both strains with OPH was tested. In order to distinguish the strains, expression vectors containing the genes for the fluorescent marker proteins GFP and Crimson were used. *C. glutamicum* WT (pECXT99A-*gfp*_UV_) and AROM3 (pECXT99A-*crimson*) were cultivated in 80 vol% OPH with 150 mM (NH_4_)_2_SO_4_ and 200 mM MOPS separately with a starting CDW of 0.35 g/L and also mixed in a co-culture with a starting CDW of 0.35 g/L each (absolute starting CDW was 0.7 g/L) (Figure 5).

*C. glutamicum* WT (pECXT99A-*gfp*_UV_) grew fast for 2 h before growth ceased at a CDW of 1.4 ± 0.1 g/L. Glucose was not exhausted until the end of the cultivation. Strain AROM3 (pECXT99A-*crimson*) showed a complex growth pattern and reached a final CDW of 4.9 ± 0.2 g/L when all the glucose was consumed after about 45 h. When both strains were cultivated together, the lag phase decreased and the growth rate increased compared to AROM3 (pECXT99A-*crimson*) alone, but growth stopped at 3.2 ± 0.2 g/L CDW even though glucose was still present in the medium (Figure 5A). Fluorescence analysis by flow cytometry (Figure 5B) revealed that about 70% of the cells showed GFP fluorescence. In conclusion, WT actively alters the medium, thereby inhibiting both its own growth and that of AROM3.

#### 3.2.2 Overcoming growth inhibition by adjusting the media preparation

**FIGURE 6:**
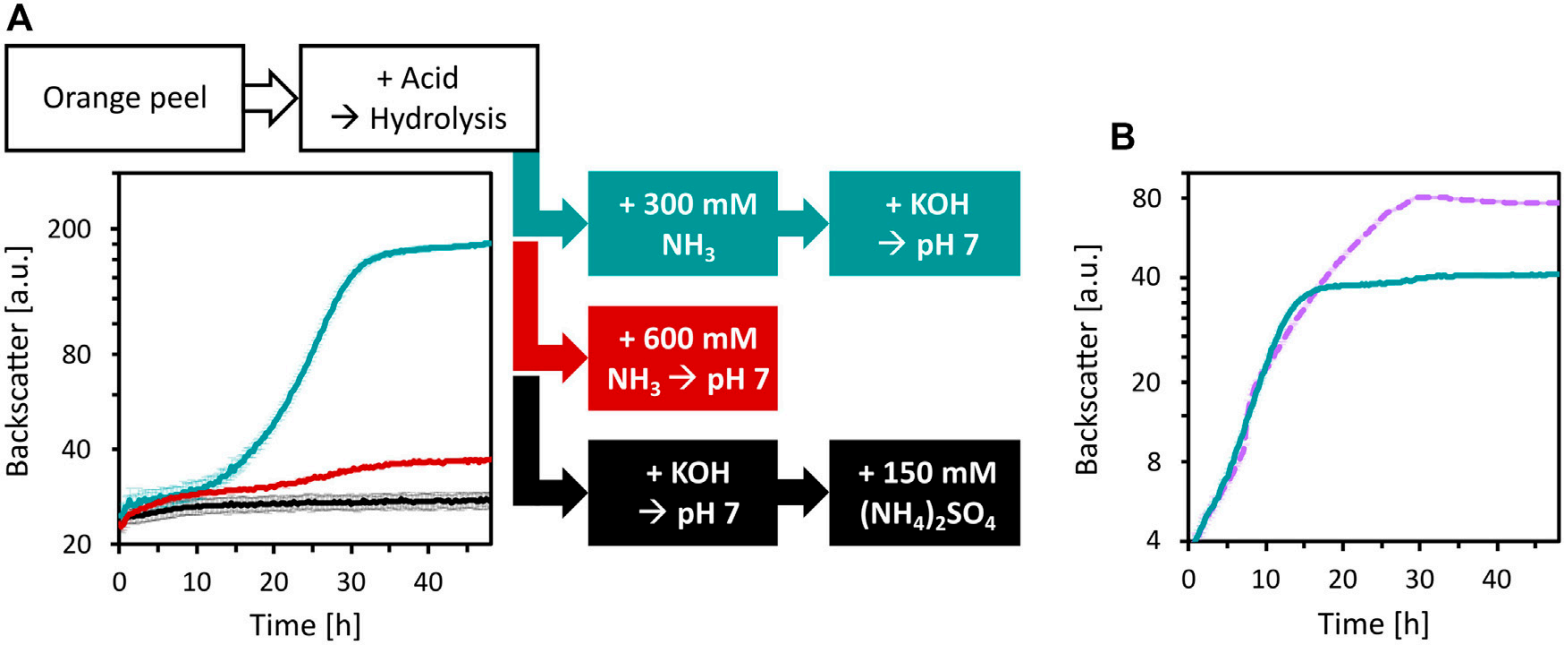
Improvement of OPH medium by changing neutralization agent and buffer. **(A)** Influence of OPH neutralization and nitrogen supplementation on growth of *C. glutamicum*. *C. glutamicum* WT (pECXT99A-*gfp*_UV_) was cultivated in OPH after pH adjustment with KOH and addition of (NH_4_)_2_SO_4_ (black), with NH_3_ (red), or with 300 mM NH_3_ and KOH (turquoise). 80 vol% OPH were supplemented with 200 mM MOPS. **(B)** Comparison of MOPS (turquoise) and phosphate buffer (dashed, purple) for the growth of *C. glutamicum* in OPH. The pH of the OPH used here was neutralized with 300 mM NH_3_ and KOH, and 80 vol% was supplemented with 200 mM buffer; the OPH with phosphate buffer supplementation is named OPH-NH_3_ medium. *C. glutamicum* WT (pECXT99A-*gfp*_UV_) was cultivated in the BioLector microcultivation system and all values and error bars represent the means and standard deviations of triplicate cultivations.

To enable the growth of *C. glutamicum* WT and its derived strains, media adjustments were tested. Since the deletion of *vdh* and *ldhA* abolished HMFCA and ʟ-lactic acid formation, the base used for pH neutralization after acid hydrolysis with H_2_SO_4_ and the choice of the buffer system were targeted. First, three ways to neutralize OPH and to provide high N loading were compared: i) addition of 150 mM (NH_4_)_2_SO_4_ (corresponds to 300 mM N) and pH neutralization with KOH (as described previously), ii) pH neutralization with NH_3_, or iii) addition of 300 mM NH_3_ followed by pH neutralization with KOH (Figure 6A). Of these treatments, the addition of 300 mM NH_3_ followed by neutralization with KOH was superior compared to the other treatments. Compared to the previously used OPH medium i), this treatment pH neutralizes OPH and provides sufficient N source, while reducing sulfate concentration.

Although 200 mM of MOPS was used as a buffer, shifts to acidic pH values during cultivation were observed (Figure 5A and data not shown). Therefore, buffering with either 200 mM MOPS or 200 mM potassium phosphate was compared for cultivation of *C. glutamicum*. Phosphate buffering was better than MOPS buffering and *C. glutamicum* grew to a CDW of 11.6 ± 0.8 g/L within 30 h (Figure 6B). OPH with 300 mM NH_3_, neutralized with KOH and buffered with 200 mM potassium phosphate was named OPH-NH_3_ medium (its elemental composition and comparison with CGXII medium is listed in the Supplementary Table S3). The OPH-NH_3_ medium was used in the following experiments.

### 3.3 Amino acid production on orange peel hydrolysate

**FIGURE 7:**
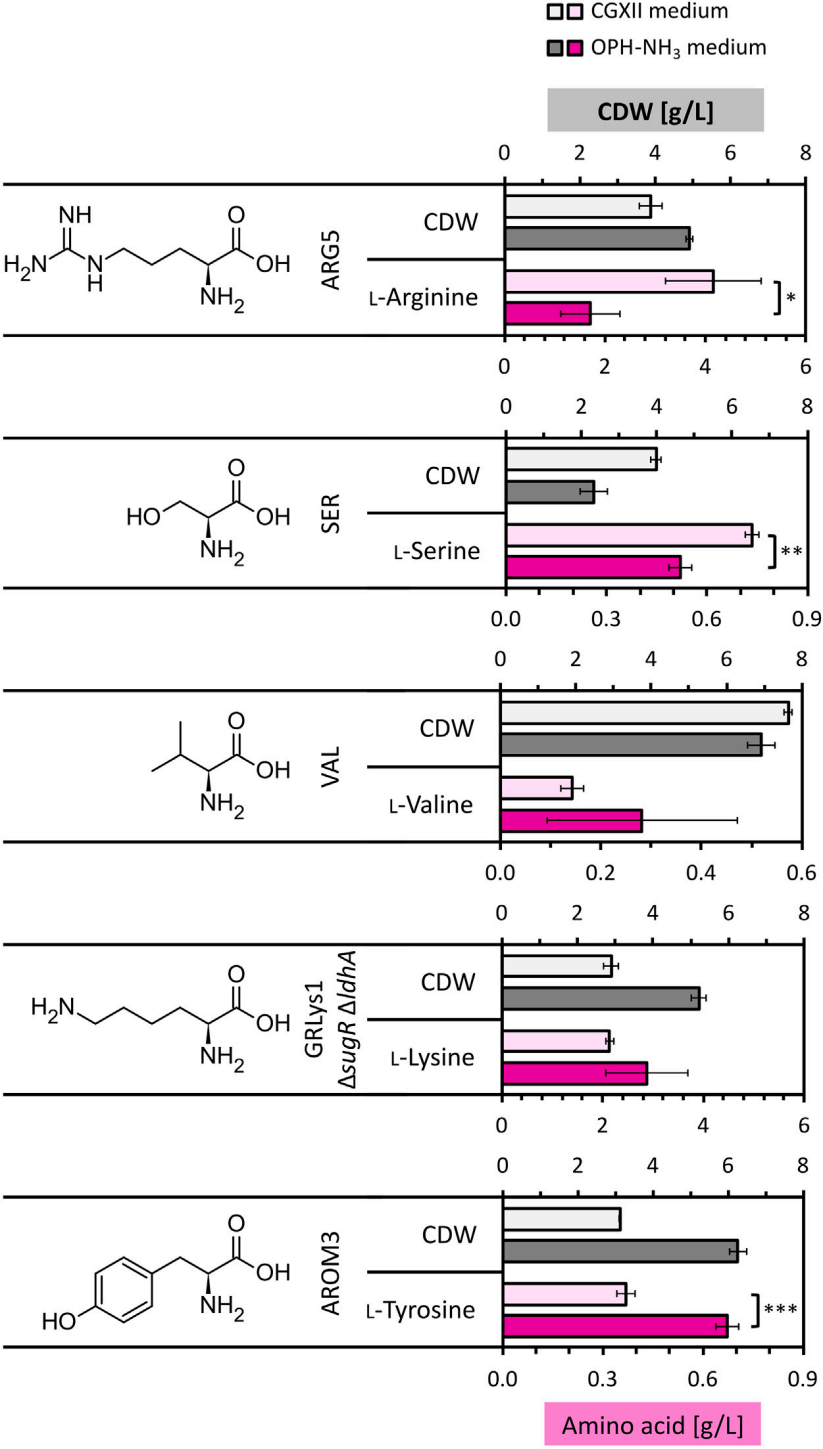
Amino acid production by *C. glutamicum* strains in OPH-NH_3_ medium. *C. glutamicum* strains ARG5, VAL1, SER1, GRLys1 Δ*sugR* Δ*ldhA* and AROM3 were cultivated in 80 vol% OPH with addition of 300 mM NH_3_ and supplemented with 200 mM phosphate buffer (dark gray and dark pink) or in CGXII medium containing 14.5 g/L glucose (light gray and light pink) for comparison. Cultivation was done in the BioLector microcultivation system for 72 h. Values and error bars of CDW (gray) and produced amino acids (pink) (the initial amino acid concentration in the medium was subtracted from the measured concentration after cultivation) represent means and standard deviations of triplicate cultivations.

The optimized OPH-NH_3_ medium was tested for amino acid production by different metabolically engineered *C. glutamicum* strains. Categorization of the metabolic precursors for amino acid biosynthesis guided our strain selection: ʟ-arginine (α-ketoglutarate) using strain ARG5 (Jensen et al., 2015), ʟ-lysine (oxaloacetate/aspartate) by GRLys1 Δ*sugR* Δ*ldhA* (Pérez-García et al., 2016), ʟ-serine (3-phosphoglycerate) by SER1 (Stolz et al., 2007), ʟ-valine (pyruvate) using strain VAL1 (Lange et al., 2003), and ʟ-tyrosine (shikimate) by strain AROM3 (Kurpejović et al., 2023). The strains were cultivated in OPH-NH_3_ medium [80 vol% OPH with 300 mM NH_3_ (added before pH neutralization with KOH) and 200 mM phosphate buffer]. For comparison, the strains were also cultivated in CGXII medium with the same glucose concentration as in OPH-NH_3_ medium, namely, 14.5 g/L (Figure 7).

ʟ-Lysine and ʟ-valine production by *C. glutamicum* strains GRLys1 Δ*sugR* Δ*ldhA* and VAL1, respectively, did not differ significantly between the two media, with 2.14 ± 0.07 g/L and 0.14 ± 0.02 g/L produced on CGXII compared to 2.87 ± 0.82 g/L and 0.28 ± 0.19 g/L produced on OPH-NH_3_ medium, respectively. Strains ARG5 and SER1 showed a decreased performance on OPH-NH_3_ medium with 1.70 ± 0.59 g/L ʟ-arginine and 0.52 ± 0.03 g/L ʟ-serine produced, respectively, compared to CGXII medium, on which 4.15 ± 0.95 g/L ʟ-arginine and 0.74 ± 0.02 g/L ʟ-serine where produced. Notably, the production of the aromatic amino acid ʟ-tyrosine with a titer of 0.67 ± 0.03 g/L was significantly increased in OPH-NH_3_ medium compared to the 0.37 ± 0.03 g/L ʟ-tyrosine produced in CGXII, with an about doubled titer reached on OPH. Thus, OPH-NH_3_ medium successfully supported the growth and amino acid production of all the tested engineered *C. glutamicum* strains.

## 4 Discussion

In this study, orange peel hydrolysate was employed as a sustainable feedstock for the cultivation of *C. glutamicum*. With addition of NH_3_ and a buffer, OPH could be used as a complete medium. Successful amino acid production on the developed OPH-NH_3_ medium was demonstrated for five engineered *C. glutamicum* strains, resulting in the production of ʟ-valine (0.28 ± 0.19 g/L), ʟ-tyrosine (0.67 ± 0.03 g/L), ʟ-serine (0.52 ± 0.03 g/L), ʟ-arginine (1.70 ± 0.59 g/L), and ʟ-lysine (2.87 ± 0.82 g/L).

*C. glutamicum* stands out as a key microorganism for the production of bulk amino acids at the multi-million ton scale. Industrial fermentation by *C. glutamicum* relies mainly on glucose, fructose, and sucrose derived from molasses or starch hydrolysates, although the carbon source is the main driver of the operational expenditure (OPEX) (Eggeling and Bott, 2015). Using waste materials as a nutrient source can provide an inexpensive feedstock while eliminating the disposal of waste. Waste materials such as grass juice (Neuner et al., 2013), rice or wheat straw (Gopinath et al., 2011; Werner et al., 2023) have been tested as alternative feedstocks for amino acid production by *C. glutamicum*. While aquaculture side stream (Schmitt et al., 2023) was supplemented to CGXII medium in addition to glucose to increase the carbon source and thus boost production, other sources such as hazelnut husk hydrolysate (Pakalın et al., 2023), rice straw (Sasikumar et al., 2021), or sorghum hydrolysate (Sasaki et al., 2019) could completely replace the carbon source. Notably, as shown here, OPH not only replaced the carbon source, but also most of the other medium components required for cultivation of *C. glutamicum*. When supplemented with NH_3_ and buffered, OPH could replace all 14 different components of the CGXII minimal medium. The additional buffer system used here for the cultivation of *C. glutamicum* was required to maintain pH and might be replaced by automated pH control in a fermenter system. Thus, besides orange waste, the preparation of this medium requires only the additional raw materials H_2_SO_4_, NH_3_ and KOH, which successfully demonstrates the substitution of numerous ingredients by food waste, creating a simple, low-cost medium.

As observed with many hydrolysates, e.g., a hydrolysate of wheat side stream concentrate from industrial starch production (Burgardt et al., 2021), growth is inhibited at higher hydrolysate concentrations. This impaired growth behavior is probably caused by growth inhibiting components which are formed or released during acid treatment of the lignocellulosic material. These compounds can be classified into three categories: furan derivatives, weak organic acids and phenolic compounds, which are formed as a result of dehydration of pentose and hexose, hydrolysis of acetyl groups, or breakdown of lignin components, respectively (Jönsson et al., 2013). The high digestibility of orange waste due to its low lignin content and porous matrix distinguishes it from crop residues or hardwoods (De la Torre et al., 2019b). For orange peels, it is reported that reducing the acid concentration to 0.5 vol% not only increases the glucose yield but also decreases the concentration of the growth inhibitor HMF (Oberoi et al., 2010; Ayala et al., 2021). Thus, orange peel is highly suitable for hydrolysis and subsequent use in microbial cultivation, but also the OPH used here for cultivation of *C. glutamicum* could be further improved by adjusting the hydrolysis parameters.

With about 2.3 g/L HMF, the accumulation of this furfural derivative in the OPH used here is not negligible. High HMF concentrations restrict the usability of acid–pretreated lignocellulosic hydrolysates, hence understanding and exploiting the detoxification mechanism is of great interest. Many organisms, such as *E. coli* or *S. cerevisiae*, detoxify furfural and HMF by reducing the aldehyde group to the alcohol, forming furfuryl alcohol and BHMF, respectively (Boopathy et al., 1993; Liu et al., 2008; Miller et al., 2010). In addition to reducing the furfural derivatives, *C. glutamicum* is also able of oxidizing furfural and HMF to their respective acids, furoic acid (Tsuge et al., 2014) and HMFCA (Zhou et al., 2019), but does not further degrade these compounds. The enzyme(s) responsible for the synthesis of furoic acid and HMFCA have not yet been described in *C. glutamicum*. In this study, we showed that HMFCA formation was reduced by 97%, when *vdh* was deleted in *C. glutamicum* WT (Figure 5), indicating that the encoded vanillin dehydrogenase (EC 1.2.1.67) is the main enzyme being responsible for the oxidation of HMF to its acid in *C. glutamicum*. Besides vanillin, the aldehyde dehydrogenase VDH shows catalytic activity towards a broad substrate range, including 3,4-dihydroxybenzaldehyde, cinnamaldehyde, syringaldehyde, and others (Ding et al., 2015). As shown by Ding et al., the *vdh* deletion strain of *C. glutamicum* retained a partial ability to grow on vanillin. This suggests the presence of alternative VDH(s), which is consistent with our observation, that a small amount of HMFCA was still formed by *C. glutamicum* Δ*vdh*. The vanillin dehydrogenase VDH1 from *Comamonas testosteroni* has also been shown to oxidize HMF to HMFCA (Zhang et al., 2020), as has the aldehyde dehydrogenase ALDH70140 from *Pseudomonas aeruginosa* (Chang et al., 2023). However, both of these enzymes share less than 40% sequence identity with the VDH from *C. glutamicum* (Supplementary Table S4). In other organisms, such as *Cupriavidus basilensis*, the FAD-dependent oxidoreductase HMfH (EC 1.1.3.47) has been described to be able to oxidize HMF to HMFCA; however, HMfH further converts HMFCA to 2,5-furandicarboxylic acid (Koopman et al., 2010) and thus greatly differs from the VDH_*C.g.*_ described here.

The high amount of the acid HMFCA formed by WT is also consistent with the acidification of the medium, which was observed for WT, but not for the *vdh* deficient strain AROM3 (Figure 5). Different genetic modifications, among them the deletion of *vdh*, were identified to reduce growth inhibition by OPH. Additive beneficial effects by replacement of *vdh* by P_*ilvC*_-*aroG*_*Ec*_^D146N^ and further deletions of the genes *ldhA*, cg2663–cg2673, and cg2674–cg2686 were identified (Figure 3), which was reflected not only in increased growth rates but also in reduced residual glucose concentrations after cultivation. Co-cultivation of the strains WT and AROM3 revealed further that the WT altered the OPH-NH_3_ medium, which inhibited both strains (Figure 5). Since pH homeostasis in *C. glutamicum* is effective in the range of 6-9, media acidification below pH 6 impairs growth (Follmann et al., 2009) and can thus be identified as one of the factors causing growth differences between WT and AROM3. Along with the contribution of *vdh* to the medium acidification via the formation of acidic detoxification products (mainly HMFCA, but probably also vanillic acid, or 3,4-dihydroxybenzoic acid), also the *ldhA*-encoded ʟ-lactate dehydrogenase (Ldh), likely contributed to medium acidification via ʟ-lactic acid secretion. Typically, ʟ-lactic acid accumulates transiently as it is re-catabolized by quinone-dependent ʟ-lactate dehydrogenase (encoded by *lldD*) (Stansen et al., 2005). As the deletion of *ldhA* abolishes transient ʟ-lactic acid accumulation (Stansen et al., 2005), a growth advantage of strains lacking *ldhA* (Δ*ldhA* and ARO02) can be explained despite the absence of ʟ-lactic acid at the end of growth. Additionally, weak acids are known to uncouple the transmembrane pH gradient (McLaughlin and Dilger, 1980), including lactic acid (Axe and Bailey, 1995), which may also apply to the acid HMFCA. Uncoupling of the pH gradient impairs the ATP generation via transmembrane ATPase, which in turn impairs growth. Consequently, both medium acidification and uncoupling effects by formed ʟ-lactic acid and HMFCA are potential explanations for the growth inhibition observed in WT and would explain the benefit of *ldhA* and *vdh* gene deletions.

Since several gene deletions contributed to the alleviation of the growth inhibition, and complete glucose consumption was only observed when all four modifications were combined, the underlying reasons are probably also multiple. ʟ-Lactic acid formation by Ldh requires NADH, as does the detoxification of furfural and HMF to their alcohols. Tsuge et al. (2014) showed that both the NADH/NAD^+^ and the NADPH/NADP^+^ ratios were dramatically decreased (by 96% and 73%, respectively) in *C. glutamicum* by adding 20 mM furfural to the medium. One NADPH-specific dehydrogenase contributing to furfural detoxification was identified to be encoded by cg0400 (designated as *fudC*) (Tsuge et al., 2016); however, crude extract of the deletion strain still had furfural dehydrogenase activity under both NADPH and NADH consumption. *C. glutamicum* thus possesses further unidentified furfural dehydrogenases. One gene encoding a putative dehydrogenase can also be found among the genes cg2673–2686, namely, cg2685, for which the function is still unknown. Another enzyme related to NADH consumption is encoded by cg2674, namely, AhpD. Although it is not NADH dependent itself, oxidized AhpD is reduced with electrons from the Lpd/SucB/NADH pathway (Su et al., 2019). Hong et al. (2019) showed that the NADH/NAD^+^ ratio is increased in *C. glutamicum* Δcg2674 compared to WT, making its deletion a putative benefit in the presence of furfural and HMF.

The presence of inhibiting components and nutrients affects not only growth ability but also production performance. The decreased ratio of NADPH/NADP^+^ upon detoxification of furfural and HMF (Tsuge et al., 2014) may perturb production of arginine, the amino acid with the highest biosynthetic NADPH demand (Zhan et al., 2019), more than production of tyrosine. Moreover, nutrient supply also influences amino acid synthesis, such as the availability of nitrogen. Xu et al. (2020) discovered that the expression of enzymes related to tyrosine synthesis is upregulated, while gene expression of most arginine-related enzymes is downregulated under high nitrogen concentrations (300 mM NH_3_) compared to low nitrogen concentrations (3 mM NH_3_). Possibly, these hitherto unknown underlying regulatory mechanism(s) may explain why tyrosine production was increased with OPH-NH_3_ medium as compared to regular minimal medium, whereas arginine production was reduced.

Unlike many other studies, this work established the use of OPH for the production of nitrogenous compounds, namely amino acids. As the multi-billion USD amino acid market is steadily rising (Wendisch, 2020), amino acid production is a promising and lucrative implementation of OPH. In the future, OPH and related hydrolysates may be used to produce other nitrogenous target compounds, such as diamines, as precursors for polyamide bioplastics (Choi et al., 2023).

## Data Availability

The original contributions presented in the study are included in the article/Supplementary Material, further inquiries can be directed to the corresponding author.
