# Supplementary material for: Utilization of orange peel waste for sustainable amino acid production by Corynebacterium glutamicum
Source: Front Bioeng Biotechnol. 2024 Jul 10;12:1419444. doi: 10.3389/fbioe.2024.1419444 (PMC11266056; doi:10.3389/fbioe.2024.1419444)
Supplement: Supplementary file 1 [file DataSheet1.pdf]

Supplementary Material

1 Supplementary Figures

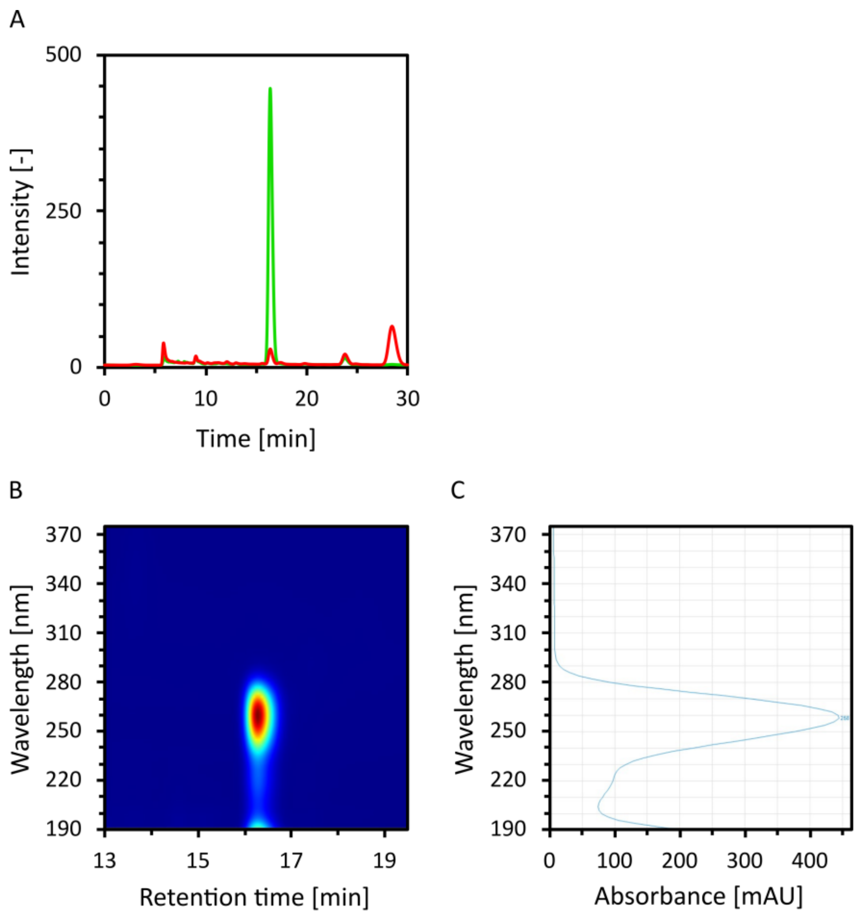

Supplementary Figure S1. HPLC measurement for the detection of HMFCA in supernatant samples after cultivation in OPH. (A) Chromatogram of HPLC measurement (detection with DAD at 260 nm) of supernatant samples after cultivation of *C. glutamicum* WT (shown in green) and AROM3 (shown in red) in 80 vol-% OPH with 150 mM $(NH_4)_2SO_4$ and 200 mM MOPS and **(B)** exemplary isoabsorbance plot (around retention time 16.4 min) for supernatant of WT cultivation with the corresponding spectrum at retention time 16.4 min, showing that the absorbance maximum is at 258 nm **(C)** and fits to the retention time and spectrum of the HMFCA standard (measurement as described in Material and Methods for measurement of HMFCA; detection with DAD at 260 nm, retention time of HMFCA: 16.4 min).

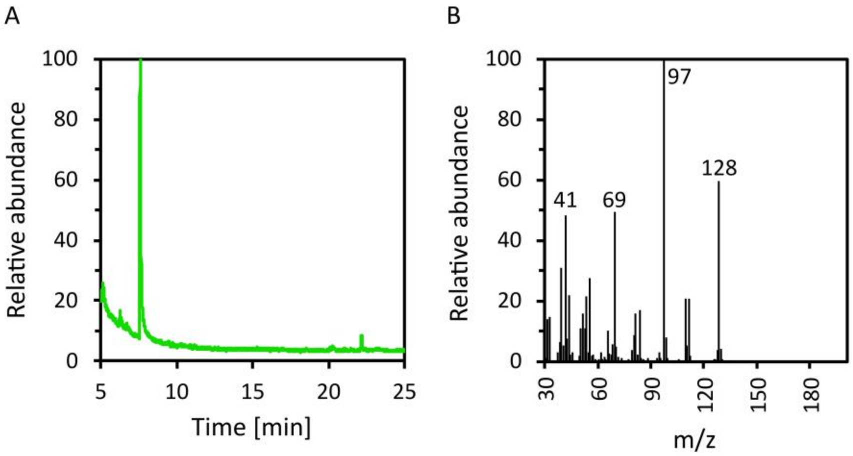

Supplementary Figure S2. GC-MS measurement for identification of BHMF after cultivation in OPH. (A) Exemplary chromatogram of GC-MS measurement of a supernatant sample after cultivation of *C. glutamicum* WT in 80 vol-% OPH with 150 mM $(NH_4)_2SO_4$ and 200 mM MOPS and **(B)** the mass spectrum corresponding to the peak with the highest abundance (at a retention time of 7.5 min) fitting to the expected mass spectrum of BHMF (measurement as described in Material and Methods).

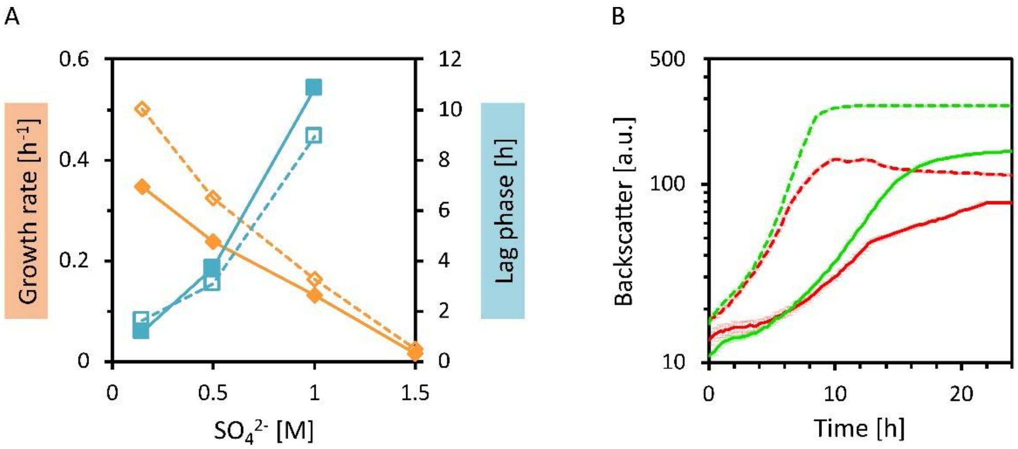

Supplementary Figure S3. Growth of *C. glutamicum* WT and AROM3 cultivated with increasing Na_2SO_4 concentrations or with addition of sorbitol. (A) The maximum specific growth rates (orange diamonds), as well as the lag phases (blue squares) are depicted for WT (dashed line and empty symbols) and AROM3 (solid line and filled symbols) for cultivation in CGXII medium (which contains 150 mM $(NH_4)_2SO_4$) plus 20 g/L glucose with addition of different concentrations of Na_2SO_4 to test final concentrations of SO_4^{2-} of 0.15, 0.5, 1 and 1.5 M in single cultivations. **(B)** The two strains WT (green lines) and AROM3 (red lines) were grown in CGXII medium plus 20 g/L glucose with (solid line) or without (dashed line) supplementation of 1 M sorbitol. Values and error bars represent means and standard deviations of triplicate cultivations.

2 Supplementary Tables

Supplementary Table S1: Amino acids in OPH prepared as detailed in Materials and Methods.

	Amino acid [g/L]
**Aspartate**	0.705 ± 0.034
**Glutamate**	0.052 ± 0.002
**Asparagine**	0.037 ± 0.002
**Serine**	0.110 ± 0.005
**Glycine/ Threonine**	0.025 ± 0.001
**Arginine**	0.069 ± 0.003
**Tyrosine**	0.034 ± 0.001
**Alanine**	0.092 ± 0.004
**Valine**	0.024 ± 0.001
**Phenylalanine**	0.047 ± 0.002
**Isoleucine**	0.015 ± 0.001
**Leucine**	0.048 ± 0.002
**Lysine**	0.030 ± 0.002
**Prolin**	0.319 ± 0.045
**Total**	1.608 ± 0.057

Supplementary Table S2: Gene deletions that contribute to an improved growth rate in OPH with $(NH_4)_2SO_4$ and MOPS. Cg number, gene name and function of the encoded protein according to Baumgart et al. (2018).

	Cg no.	NCgl Synonym	Gene name	Function of encoded protein	Functional categorization
**Δcg2663-2673**	cg2664	NCgl2341		putative type-IV restriction endonuclease	DNA replication, recombination, repair, and degradation
	cg2665	NCgl2342		hypothetical protein	Unknown function
	cg2666	NCgl2343		hypothetical protein	Unknown function
	cg2667			hypothetical protein	Unknown function
	cg2668		*crtI2-2*	phytoene desaturase C-terminal fragment, putative pseudogen	Transport and metabolism of further metabolites
	cg2670	NCgl2346	*crtI2-1*	phytoene dehydrogenase desaturase N-terminal fragment, putative pseudogen	Transport and metabolism of further metabolites
	cg2672	NCgl2347	*crtB2*	phytoene synthetase	Transport and metabolism of further metabolites
	cg2673	NCgl2348		putative permease of the major facilitator superfamily	General function prediction only
**Δcg2674-2686**	cg2674	NCgl2349	*ahpD*	putative alkylhydroperoxidase AhpD-family core domain	General function prediction only
	cg2675	NCgl2350		putative ATPase component of ABC-type transport system, contains duplicated ATPase domains	General function prediction only

cg2676	NCgl2351		putative ABC-type dipeptide/oligopeptide/nickel transport systems, permease component	General function prediction only
cg2677	NCgl2352		putative ABC-type dipeptide/oligopeptide/nickel transport system, permease component	General function prediction only
cg2678	NCgl2353		putative ABC-type dipeptide/oligopeptide/nickel transport systems, secreted component	General function prediction only
cg2679	NCgl2354		hypothetical protein	Unknown function
cg2680	NCgl2355	*argD2*	putative aminotransferase class-III, pyridoxal-phosphate dependent	Amino acid transport and metabolism
cg2683	NCgl2356		hypothetical protein, conserved	Unknown function
cg2684	NCgl2357		putative membrane protein DedA-family, conserved	Unknown function
cg2685	NCgl2358		putative short chain dehydrogenase/reductase	General function prediction only
cg2686	NCgl2359		putative transcriptional regulator, TetR-family	Signal transduction mechanisms
cg2953	NCgl2578	*vdh*	vanillin dehydrogenase (EC:1.2.1.64)	Carbon source transport and metabolism
cg3219	NCgl2810	*ldhA*	NAD-dependent L-lactate dehydrogenase	Anaerobic metabolism

Supplementary Table S3: Elementary composition of OPH-NH_3 medium (determined by Eurofins Agraranalytik Deutschland GmbH (Jena, Germany) as stated in Material and Methods) compared to CGXII medium (pH 7) containing 20 g/L glucose (as described by Eggeling and Bott (2005)).

	CGXII medium with 20 g/L glucose [g/L]	OPH-NH_3 Medium [g/L]
**C**	9.00	23
**N**	6.60	3.15
**P**	0.41	11.21
**K**	4.22	14.32
**S**	4.91	5.42
**Ca**	0.004	0.16
**Mg**	0.024	0.10
**Mn**	3.25×10^{-4}	2.90×10^{-4}
**Zn**	2.27×10^{-5}	2.11×10^{-4}
**Cu**	7.96×10^{-6}	9.93×10^{-5}
**Ni**	4.94×10^{-7}	n.d.
**Fe**	2.01×10^{-4}	n.d.

Supplementary Table S4: Comparison of enzymes with the ability to oxidize HMF to HMFCA. Sequence alignments were done using MUSCLE (MUltiple Sequence Comparison by Log-Expectation) provided by EMBL-EBI (Madeira et al., 2022).

Enzyme	VDH$_{C.g.}$	VDH1$_{C.t.}$	ALDH70140$_{P.a.}$
**Organism**	*Corynebacterium glutamicum*	*Comamonas testosteroni*	*Pseudomonas aeruginosa*
**Percent Identity to VDH$_{C.g.}$**	100	37.55	34.18
**Sequence**	VTATFAGIDATKHLIGGQWVEG<br>NSDRISTNINPYDDSVIAESKQ<br>ASIADVDAAYEAAKKAQAEWAA<br>TPAAERSAIIYRAAELLEEHRE<br>EIVEWLIKESGSTRSKANLEIT<br>LAGNITKESASFPGRVHGRISP<br>SNTPGKENRVYRVAKGVVGVIS<br>PWNFPLNLSIRSVAPALAVGNA<br>VVIKPASDTPVTGGVIPARIFE<br>EAGVPAGVISTVAGAGSEIGDH<br>FVTHAVPKLISFTGSTPVGRRV<br>GELAINGGPMKTVALELGGNAP<br>FVVLADADIDAAAQAAAVGAFL<br>HQGQICMSINRVIVDAAVHDEF<br>LEKFVEAVKNIPTGDPSAEGTL<br>VGPVINDSQLSGLKEKIELAKK<br>EGATVQVEGPIEGRLVHPHVFS<br>DVTSDMEIAREEIFGPLISVLK<br>ADDEAHAAELANASDFGLSAAV<br>WSKDIDRAAQFALQIDSGMVHI<br>NDLTVNDEPHVMFGGSKNSGLG<br>RFNGDWAIEEFTTDRWIGIKRS	MIEQKMLIAGQECAASNGAVFE<br>RKNPLDGSVATRAPAATTEDAI<br>RACDAAAAAFPAWSQLGPNARR<br>AMLMKASQALEAKGEAIAAAMA<br>AETGASGIWAGFNVHLAASMLL<br>EAASLTTQINGEIIPSDVPGSL<br>AMAVRQPAGVVLGIAPWNAPVI<br>LAVRSISTALACGNTVILKGSE<br>LCPATHGLIIEALQDAGLPAGV<br>VNFVTNAPADAGSVVEAIVAHP<br>AVRRVSFTGSTRVGRIIGQTCA<br>KHLKPALLELGGKAPFLVLDDA<br>DIDAAVSAATFGAFANSGQICM<br>STERFVVDNKVADEFIAKFAAK<br>ARSLPLGDPRKGPVVLGSVVDL<br>ATVERCNAMIDDALAKGGKLVC<br>GGKAESTLMPATLIDHVTPAMR<br>IFHEESFGPVKGIVRVNGEEEA<br>IATANDNEFGLSSAVFTRDTAR<br>GWRVAARIEAGICHINGPTVHD<br>EAQMPFGGVKASGYGHFGGQQG<br>INAFTETRWVTMQTAERHYPF	MTTLTRADWEARAKDLKIEGRA<br>FVNGEYSNAASGETFDCLSPVD<br>GRFLAKVASCDLADAEQAVKVA<br>RNAFDSGAWSRLAPAKRKQAMI<br>RFADLLLENAEELALLETLDMG<br>KPISDSLHIDVASAANSLRWSA<br>EAIDKIYDEVAATPHAELGLVT<br>REPVGVVAAIVPWNFPLLMSCW<br>KLGPALATGNSVILKPSEKSPL<br>TAIRIAQLAVEAGIPKGVFNVL<br>PGYGHTVGKALALHMDVDTLVF<br>TGSTKIAKQLMVYAGESNMKRV<br>WLEAGGKSPNIVFADAPDLKAA<br>AEAAAGAIAFNQGEVCTAGSRL<br>LVERSIKDKFLPLVIEALKGWK<br>PGNPLDPETNVGALVDTQQMNT<br>VLSYIEAGHNDGAKLVAGGKRT<br>LEETGGTYVEPTIFDGVTNAMK<br>IAREEIFGPVLSVIEFEDAEEA<br>VRIANDTPYGLAAAVWTSNLSK<br>AHLTAKALRAGSVWVNQYDGGD<br>MTAPFGGFKQSGNGRDKSLHAF<br>DKYTELKATWIKL
**Reference**	(Ding et al., 2015)	(Zhang et al., 2020)	(Chang et al., 2023)

3 References

Baumgart, M., Unthan, S., Kloß, R., Radek, A., Polen, T., Tenhaef, N., et al. (2018). *Corynebacterium glutamicum* Chassis C1*: Building and Testing a Novel Platform Host for Synthetic Biology and Industrial Biotechnology. *ACS Synth Biol* 7, 132–144. doi: 10.1021/acssynbio.7b00261

Chang, S., Li, B., Chen, T., Zhang, L., Li, Y., He, X., et al. (2023). Engineering aldehyde dehydrogenase PaALDH70140 from *Pseudomonas aeruginosa* PC-1 with improved catalytic properties for 5-hydroxymethyl-2-furancarboxylic acid synthesis. *Biochemical Engineering Journal* 192, 108835. doi: 10.1016/j.bej.2023.108835

Ding, W., Si, M., Zhang, W., Zhang, Y., Chen, C., Zhang, L., et al. (2015). Functional characterization of a vanillin dehydrogenase in *Corynebacterium glutamicum*. *Sci Rep* 5, 8044. doi: 10.1038/srep08044

Eggeling, L., and Bott, M. (2005). *Handbook of Corynebacterium glutamicum*. CRC Press.

Madeira, F., Pearce, M., Tivey, A. R. N., Basutkar, P., Lee, J., Edbali, O., et al. (2022). Search and sequence analysis tools services from EMBL-EBI in 2022. *Nucleic Acids Res* 50, W276-W279. doi: 10.1093/nar/gkac240

Zhang, X.-Y., Ou, X.-Y., Fu, Y.-J., Zong, M.-H., and Li, N. (2020). Efficient synthesis of 5-hydroxymethyl-2-furancarboxylic acid by *Escherichia coli* overexpressing aldehyde dehydrogenases. *J Biotechnol* 307, 125–130. doi: 10.1016/j.jbiotec.2019.11.007
